# Behavioral measures of impulsivity and compulsivity in adolescents with nonsuicidal self-injury

**DOI:** 10.1017/S1092852921000274

**Published:** 2021-04-23

**Authors:** Nina M. Lutz, Samuel R. Chamberlain, Ian M. Goodyer, Anupam Bhardwaj, Barbara J. Sahakian, Peter B. Jones, Paul O. Wilkinson

**Affiliations:** 1 Department of Psychiatry, University of Cambridge, Cambridge, United Kingdom; 2 Department of Psychiatry, University of Southampton, Southampton, United Kingdom; 3 Cambridgeshire and Peterborough NHS Foundation Trust, Cambridge, United Kingdom

**Keywords:** Compulsivity, impulsivity, decision making, cognitive, adolescent, self-harm, self-injury, NSSI

## Abstract

**Background:**

Nonsuicidal self-injury (NSSI) is prevalent among adolescents and research is needed to clarify the mechanisms which contribute to the behavior. Here, the authors relate behavioral neurocognitive measures of impulsivity and compulsivity to repetitive and sporadic NSSI in a community sample of adolescents.

**Methods:**

Computerized laboratory tasks (Affective Go/No-Go, Cambridge Gambling Task, and Probabilistic Reversal Task) were used to evaluate cognitive performance. Participants were adolescents aged 15 to 17 with (n = 50) and without (n = 190) NSSI history, sampled from the ROOTS project which recruited adolescents from secondary schools in Cambridgeshire, UK. NSSI was categorized as sporadic (1-3 instances per year) or repetitive (4 or more instances per year). Analyses were carried out in a series of linear and negative binomial regressions, controlling for age, gender, intelligence, and recent depressive symptoms.

**Results:**

Adolescents with lifetime NSSI, and repetitive NSSI specifically, made significantly more perseverative errors on the Probabilistic Reversal Task and exhibited significantly lower quality of decision making on the Cambridge Gambling Task compared to no-NSSI controls. Those with sporadic NSSI did not significantly differ from no-NSSI controls on task performance. NSSI was not associated with behavioral measures of impulsivity.

**Conclusions:**

Repetitive NSSI is associated with increased behavioral compulsivity and disadvantageous decision making, but not with behavioral impulsivity. Future research should continue to investigate how neurocognitive phenotypes contribute to the onset and maintenance of NSSI, and determine whether compulsivity and addictive features of NSSI are potential targets for treatment.

## Introduction

Approximately one in five adolescents (22%) engage in nonsuicidal self-injury (NSSI), the direct and deliberate damage of body tissue without suicidal intent^1^. NSSI is most often used to temporarily relieve negative affect, although other functions—such as reducing suicidal thoughts, ending dissociative states, or eliciting social support—are common^2,3^. NSSI typically emerges in adolescents between 12 and 16 years of age and appears to resolve by young adulthood in most cases.^4,5^ Studies show that at least half of those who endorse a history of NSSI hurt themselves only a few times,^6,7^ however, a subgroup of individuals continue the behavior chronically and require clinical intervention^8^.

Dimensional approaches to psychiatry have received increasing attention in recent years, offering a new framework for understanding shared mechanisms underlying previously separate diagnostic categories^9–11^. This approach is particularly relevant to the study of NSSI which is itself a transdiagnostic behavior, common among people with a range of diagnoses (internalizing, externalizing, and personality disorders) or who do not meet any formal DSM-5 diagnostic criteria^12^. Furthermore, NSSI may share parallels with different disorders involving potentially risky reinforcing behaviors, such as substance misuse and behavioral addictions, which are thought to share neurocognitive etiology^11,13,14^. Identifying common factors which underlie these maladaptive, although potentially rewarding, behaviors could provide valuable insight into transdiagnostic intervention and prevention strategies.

The overlapping neurocognitive phenotypes “impulsivity” and “compulsivity” have become a focus of this emerging literature. Both constructs are implicated in difficulty controlling behavioral responses.^11^ Impulsivity broadly describes a pattern of rash reactions without due consideration for consequences.^15^ Many people report NSSI which fits this “impulsive” definition: the behavior is often performed quickly in response to negative emotions and without prior planning.^16,17^ However, impulsivity is a multidimensional construct^11,15^with distinct facets sharing little variance and differentially relating to mental health outcomes.^18–22^Two dimensions of impulsivity may be especially relevant to understanding NSSI. First, *behavioral disinhibition* is the relative inability to stop oneself carrying out a motor behavior (eg, NSSI).^23^ Second, *impulsive decision making* involves choosing behaviors which are less likely to have a positive outcome, but with a potentially high reward^24^—people who engage in NSSI may envision the fast reward of affect relief while discounting negative consequences, such as scarring.

Yet, while people with NSSI commonly report increased impulsivity on self-report questionnaires, they often display no differences in behavioral impulsivity on laboratory tasks.^25,26^ Two characteristics of past behavioral impulsivity studies may contribute to their inconsistent and largely negative findings. First, only a subset of people with NSSI may be characterized by impulsivity, but past studies have not considered the heterogeneity of their samples. Second, NSSI typically occurs in the context of negative emotion,^27^ yet few behavioral studies have incorporated an affective component in their neurocognitive tasks^25^. Indeed, some more recent research which has significantly related NSSI to behavioral impulsivity utilized affective measures: two studies found that people with NSSI demonstrate impaired behavioral inhibition specifically in response to negative emotional images,^28,29^ and another revealed that NSSI frequency is significantly related to impulsive decision making during negative mood induced by criticism^30^.

Although it has received less attention in the literature, heightened compulsivity may similarly contribute to NSSI. *Compulsivity* describes the tendency to engage in persistent and repetitive behaviors despite their negative consequences, coupled with a lack of sensitivity to goals and punishment^11,24^. The behavior may begin as something purposeful, carried out to obtain a desired goal—such as using NSSI to get relief from negative emotions—but through reinforcement becomes a maladaptive habit^24,31^. Many individuals with long-term repetitive NSSI describe their self-injury as an addiction, reporting an escalation of frequency and severity over time, strong and persistent self-injurious urges, and loss of control over the behavior^32,33^. People who engage in NSSI often report feelings of shame, guilt, self-disgust, and self-criticism stemming from their self-injury^34,35^. Heightened compulsivity may contribute to this difficulty disengaging from NSSI despite mounting negative consequences as the behavior becomes a conditioned response to emotional distress.

In this study, we relate computerized behavioral measures of latent impulsive and compulsive phenotypes to NSSI in a community sample of adolescents. We evaluate whether these distinct neurocognitive traits are differentially related to sporadic (max. 1-3 instances per year) vs repetitive NSSI (4 or more instances per year). Our primary hypotheses are: (1) both sporadic and repetitive NSSI will be characterized by impulsivity (emotion-relevant behavioral disinhibition and impulsive decision making), and (2) repetitive NSSI will be associated with increased compulsivity.

## Methods

### Participants

Participants were sampled from the ROOTS study which recruited n = 1185 adolescents (aged 14 at entry) from 18 secondary schools in Cambridgeshire, UK. The project commenced in 2005 as a longitudinal investigation of genetic and environmental predictors for adolescent mental illness.^36,37^ Participants here were part of a ROOTS substudy assessing neurocognitive functioning and were selected based on the presence or absence of childhood adversity before age 6 and allelic variation of the promoter region of *5-HTTLPR*, involved in the regulation of serotonergic functions within the brain.^38^ Rates of childhood adversity and possession of the homozygous short allele of *5-HTTLPR* here are representative of a general population sample^38^.

The final sample included 240 participants (114 male, 126 female) aged 15 to 17 years (Table 1). Face-to-face and questionnaire assessments at ages 14 and 17 (respectively, before and after neuropsychological testing) collected data on lifetime NSSI, demographic information, and psychopathology. At the time of neuropsychological testing (approximately age 16), participants were evaluated for recent depressive symptoms and intelligence.

Ethical approval was provided by the Cambridgeshire 2 Research Ethics Committee (reference numbers 03/302 and 09/H0308/168), following the Declaration of Helsinki. Participants and their parents provided written informed consent prior to participation.

## Materials

Participants were categorized as high or low socioeconomic status (SES) using a proxy measure based on postcodes (ACORN classification; https://acorn.caci.co.uk). At the time of neuropsychological testing, general intellectual ability was assessed via a short form of the Wechsler Intelligence Scale for Children III, UK version.^39^ The vocabulary and block design subtests were combined to produce a full IQ score. The Moods and Feelings Questionnaire (MFQ)^40^ was used to assess depressive symptoms in the past 2 weeks.

Psychiatric diagnoses were assessed at age 14 using the Kiddie Schedule for Affective Disorders, present and lifetime version,^41^ using DSM-IV criteria. In addition, participants reported NSSI history at ages 14 and 17. Lifetime NSSI was assessed via the Drug and Self-Injury Questionnaire, with established validity and reliability^42^. A yes/no question asked: *“Have you ever tried to hurt yourself on purpose without trying to kill yourself?* (*for example: things like burning, cutting or scratching yourself*)”. NSSI frequency was assessed via the multiple-choice question: “*What is the greatest number of times, in any one year, that you have tried to hurt yourself in the way described* [*in the previous question*]?” Answer choices were: “never,” “once,” “2 to 3 times in a year,” and “4 or more times in a year.” Participants were categorized based on the maximum frequency of NSSI reported at either time point. In line with previous research,^43^ sporadic NSSI was defined as maximum one to three times per year, and repetitive as four or more times per year.

## Affective Go/No-Go

**Figure 1:**
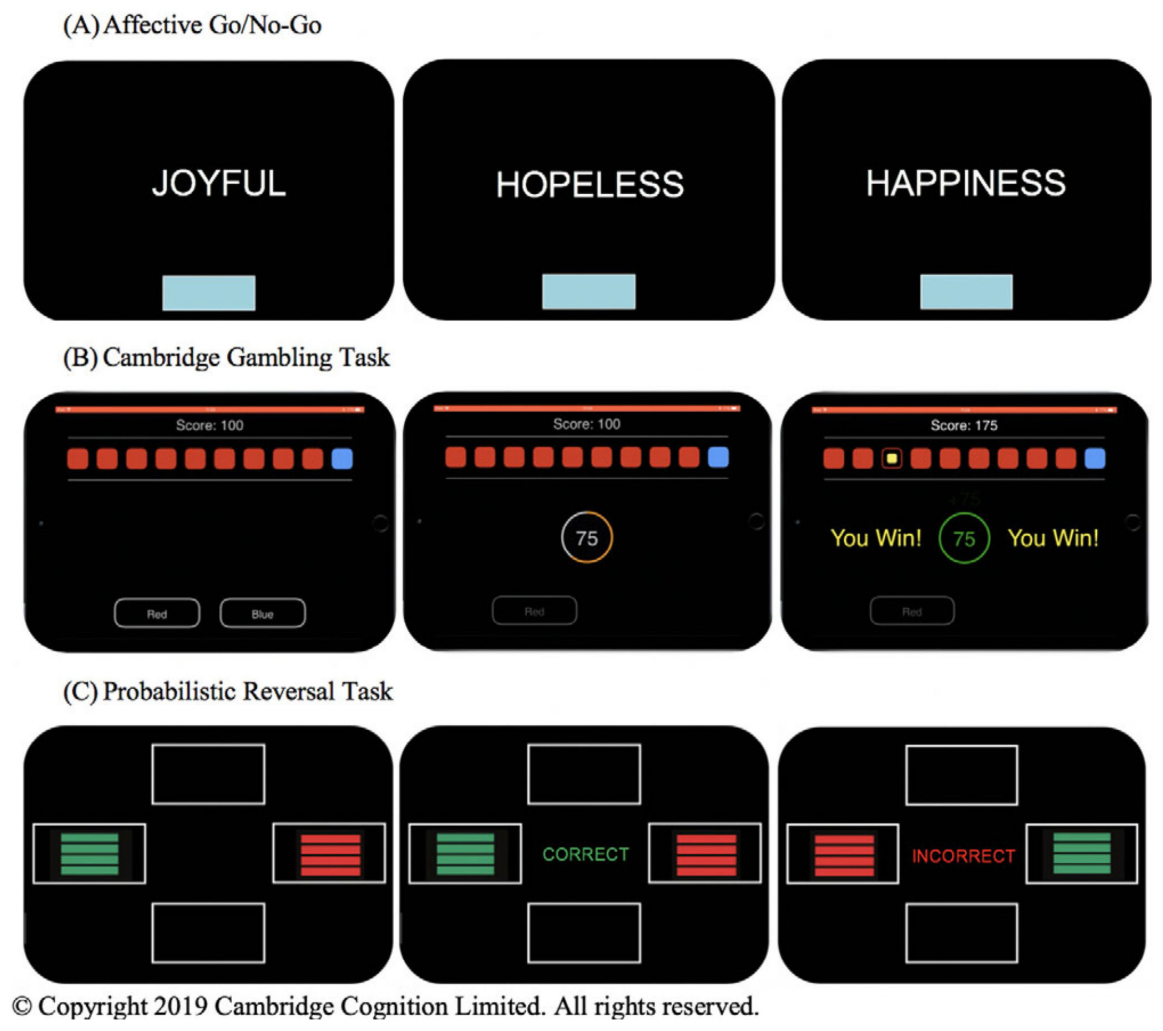
Representations of the computerized behavioral laboratory tasks used. (A) On the Affective Go/No-Go, participants are presented with a series of positive, negative, and neutral valence words and instructed to respond only to wordsofthetargetvalence while ignoringwordsofthe distractor valence. The targetand distractorvalencesvarybetween task blocks. (B) In each trial ofthe Cambridge GamblingTask, participants are presented with 10 colored boxes and told that a token is hidden underone ofthe boxes. Participants must guess which color box contains the hidden token and then bet a proportion of their total points. They gain those points if they are correct and lose them if they are incorrect. The ratio ofcolored boxes varies in each trial. (C) On the Probabilistic Reversal Task, participants are presented with two colored stimuli. They must determine through trial and errorwhich coloris “correct” and are instructed to selectthis “correct” coloreverytime, even intheface ofmisleading negativefeedback.Theyaretold the ruleswillchange at some point during the task and that they must then select the new “correct” color every time, though they are not told when this switch will occur. Both the “correct” and “incorrect” stimuli receive misleading feedback on 20% of trials. © Copyright 2019 Cambridge Cognition Limited. All rights reserved.

Behavioral inhibition was assessed via the Affective Go/No-Go (AGNG) task from the CANTAB® battery (Cambridge Cognition, Cambridge, UK).^44^ During the task, participants are rapidly presented with a series of positive, negative, and neutral valenced words on a computer screen (Figure 1). Each block contains words of one target valence and one distractor valence. Participants are instructed to press the spacebar as quickly as possible when presented with a target valence word but withhold their response when presented with a distractor valence word. The task begins with two practice blocks followed by 18 test blocks which are presented in an order which creates nine “non-shift” blocks in which the target valence remained the same as the preceding block, and nine “shift” blocks in which the target valence is changed.

Our outcome of interest was commission errors (false positive responses to distractor stimuli, ie, behavioral disinhibition). In particular, we were interested in mood-congruent attentional biases: whether the pattern of differences in commission errors between groups differed across word emotional valences (group × valence interaction), as has been shown in the only lab studies to find differences in behavioral inhibition between those with and without NSSI^28,29^.

## Cambridge Gambling Task

Impulsive decision making was assessed using the Cambridge Gambling Task (CGT) from the CANTAB® battery.^45^ In each trial, the participant is presented with 10boxes (redorblue) and told that the computer has hidden a token under one of the boxes (Figure 1). The ratio of colored boxes varies in each trial, with five possible probabilities (9:1, 8:2, 7:3, 6:4, 5:5). Participants must first decide whether the token is under a red or blue box, then bet a proportion of their total points. After placing a bet, the token is revealed and the participant either gains (if they were correct) or loses (if they were incorrect) those points. The task begins with four practice trials followed by eight test blocks of nine trials.

Three dependent measures of interest were: (1) overall proportion of points bet across trials; (2) quality of decision making, that is, the proportion of trials in which the participant chose the most likely outcome; and (3) response time (a faster response time would support that decision making was impulsive). Impulsive decision making is defined by the combination of high proportion of points bet and disproportionately choosing the risky option (poor decision making)^24^.

## Probabilistic reversal task

Compulsivity was assessed using the Probabilistic Reversal Task (PRT)^46^ consisting of a discrimination stage (40 trials) and reversal stage (40 trials). In each trial, participants are presented with two colored stimuli (Figure 1). Participants must determine through trial and error which color is usually correct and continue to select that correct color even in the face of misleading negative feedback. The color selected by the subject in the first trial becomes the “correct” color in the discrimination set and receives 80% positive feedback and 20% misleading negative feedback. Similarly, the “wrong” stimulus receives 20% misleading positive feedback and 80% negative feedback. Participants are instructed that the rules may change during the task, but are not told when it will happen. At the reversal phase, the rules are reversed and participants must abandon the previous response and choose the new correct stimulus every time. Participants are deemed to have passed each stage if they select the correct color eight times in a row.

The PRT has three dependent measures of interest: (1) perseverations, that is, the number of incorrect responses after the reversal, which signifies difficulty abandoning the previously learned response pattern despite negative feedback; (2) errors to criterion in each stage, that is the number of trials before the participant passes the stage; and (3) probability matching score, that is, how often participants abandon the correct response after receiving misleading negative feedback.^47^ Increased perseverations suggest compulsivity (ie, continuing in old habitual behavior, despite this not meeting goals).

## Statistical analysis

All analyses were conducted using STATA 12. Groups were compared on demographic and clinical variables in a series of chisquare tests and one-way ANOVAs. Task performance was analyzed across groups in series of regression analyses. Linear regressions and multilevel mixed-effects linear regressions were used for continuous dependent variables (with robust estimators if indicated). Negative binomial regressions and multilevel mixed effects negative binomial regressions were used to analyze count data, as data did not follow Poisson distributions. Multiple regressions controlled for age, sex, intelligence (IQ), and recent depressive symptoms (MFQ; Table S1). We compared NSSI groups to controls, followed by analyses of sporadic vs repetitive NSSI. To correct for multiple comparisons, the Benjamini-Hochberg procedure^48^ was used with an FDR threshold of 0.15^49^ and eight primary tests (perseveration—PRT perseveration; motor impulsivity—AGNG group × valence commissions; impulsive decision making—CGT quality of decision making and points bet; all repeated for repetitive NSSI vs control and sporadic NSSI vs control).

## Results

### Descriptive statistics

Demographic and clinical data are shown in Table 1. Overall, 29.4% of female participants and 11.4% of male participants reported lifetime NSSI. Groups did not differ on age, SES, ethnicity, or intelligence (IQ). Three participants reported current psychotropic medication use (n = 1 no-NSSI control, n = 1 sporadic NSSI, n = 1 repetitive NSSI).

### Affective Go/No-Go

Contrary to our hypothesis, neither sporadic nor repetitive NSSI was associated with increased commission errors (Table 2), whether across the task (sporadic NSSI *β* = 0.11, *p* = .29; repetitive NSSI *β* = 0.094, *p* = 0.44) or response pattern across valences (sporadic NSSI *β* = —0.09, *p* = 0.50; repetitive NSSI *β* = 0.11, *p* = 0.44). There were no significant differences in mean correct latency between groups.

### Cambridge Gambling Task

**Figure 2:**
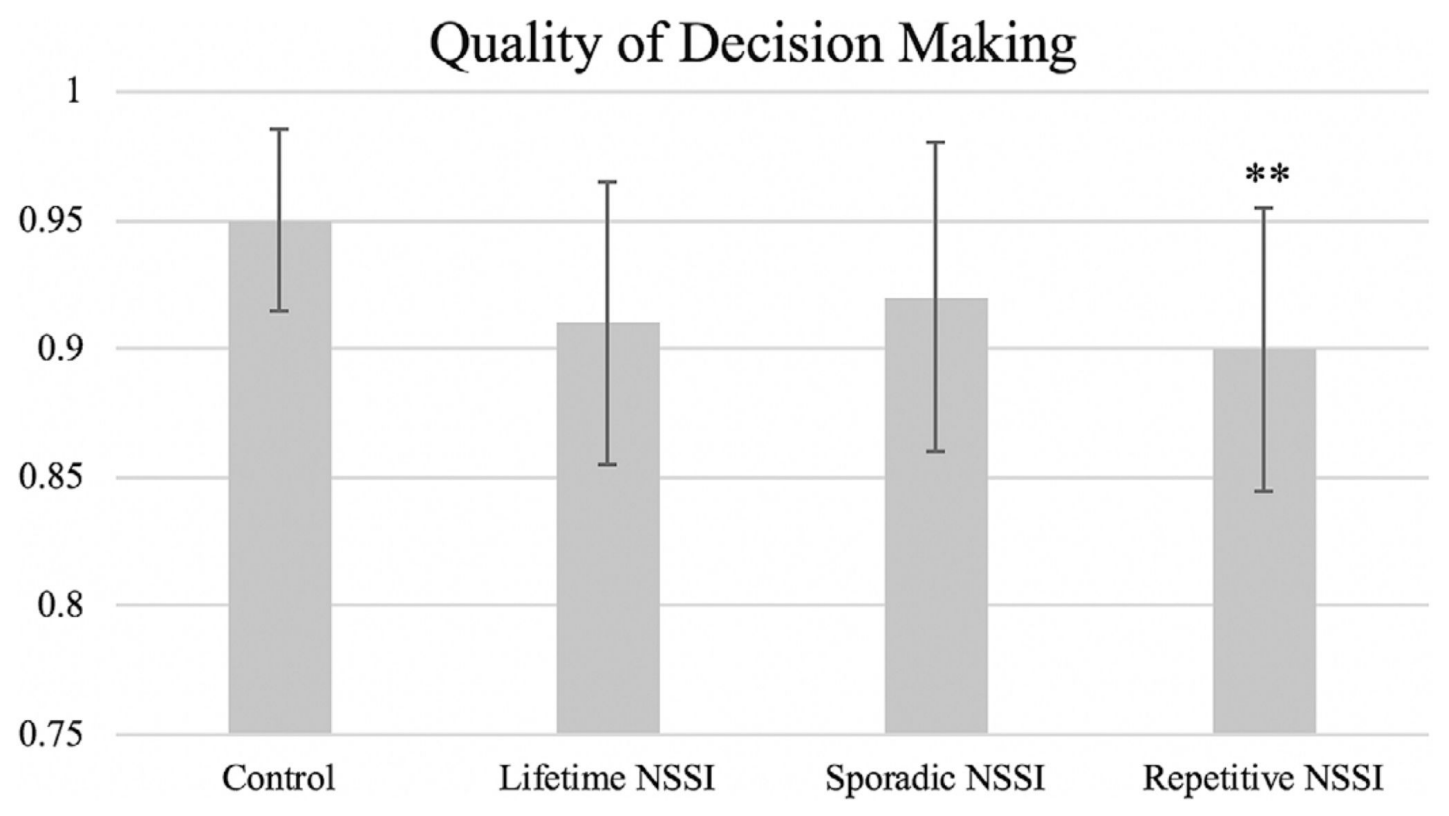
Adolescents with repetitive NSSI demonstrated significantly lower quality of decision makingthan controls on the Cambridge GamblingTask. Quality of decision making measured as the proportion of trials in which the participant selects the most likely outcome (ie, color with the greatest number of boxes). (Note: Contrast, group vs control: **p < 0.01, significant according to Benjamini-Hochberg procedure. Comparisons of sporadic vs repetitive NSSI did not reveal any significant results.)

Adolescents with repetitive NSSI (*β* = —0.06, *p* = 0.021, significant according to Benjamini-Hochberg) demonstrated significantly lower quality of decision making than controls (Table 2 and Figure 2). However, sporadic NSSI was not associated with quality of decision making (*β* = —0.03, *p* = 0.14), and neither sporadic (*β* = —0.01, *p* = 0.74) nor repetitive (*β* = 0.07, *p* = 0.06) NSSI were associated with a higher proportion of points being bet. There were no significant differences in response latency between groups.

### Probabilistic Reversal Task

**Figure 3:**
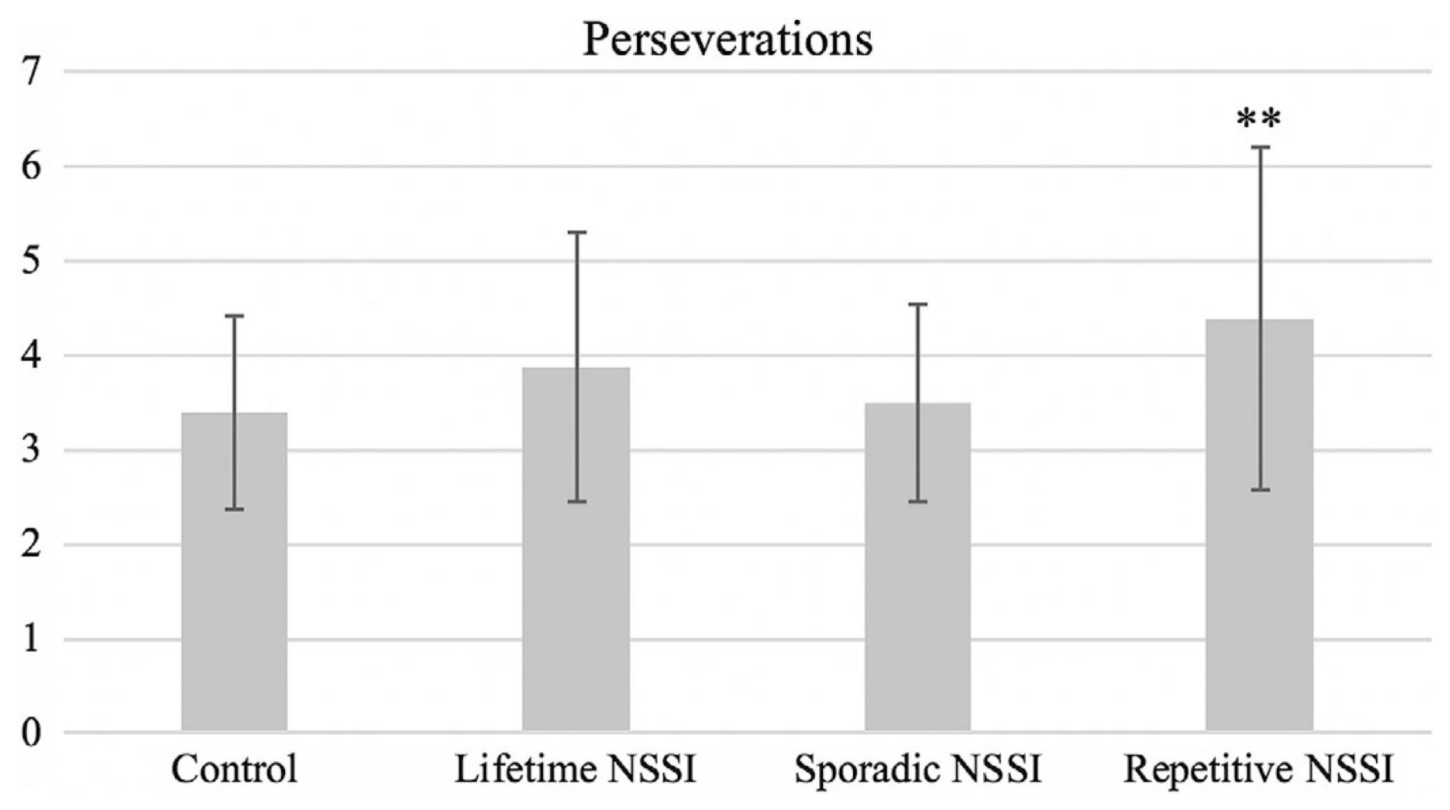
Adolescents with repetitive NSSI made significantly more perseverative errors on the Probabilistic Reversal Task than controls, signifying a pattern of compulsive responding. Perseverations are defined as successive incorrect responses following rule reversal midway through the task. (Note. Contrast, group vs control: **p < 0.01, significant according to Benjamini-Hochberg procedure. Comparisons of sporadic vs repetitive NSSI did not reveal any significant results.)

Consistent with our hypothesis, adolescents with lifetime NSSI (*β* = 0.20, *p* = 0.04), in particular repetitive NSSI (*β* = 0.36, *p* = 0.006, significant according to Benjamini-Hochberg) made significantly more perseverative errors than controls, indicating increased compulsivity (Table 2 and Figure 3). Sporadic NSSI was not significantly associated with perseverative errors (*β* = 0.08, *p* = 0.50).

## Discussion

In this study, we related NSSI to cognitive measures of impulsivity and compulsivity due to similarities between NSSI other maladaptive and reinforcing behaviors (eg, substance misuse and behavioral addictions) which are characterized by these latent neurocognitive phenotypes.^11,13^ Many people who engage in NSSI describe experiences which conceptually fit these constructs: hurting themselves with little forethought of awareness of their motives, experiencing self-injurious urges which are difficult to resist, continuing NSSI engagement despite a desire to stop, perceived loss of control over frequency and severity over time^17,32,33^. We hypothesized that both sporadic and repetitive NSSI would be associated with behavioral impulsivity, as measured by deficits in emotion-relevant behavioral inhibition and impulsive decision making, while repetitive NSSI would be associated with behavioral compulsivity.

Results partially supported these hypotheses—we found evidence of a significant association with compulsivity, but not impulsivity. Compared to no-NSSI controls, adolescents with repetitive NSSI demonstrated significantly greater perseverance on an expired set of rules in the Probabilistic Reversal Task, which can be interpreted as a neuropsychological model of compulsivity^24,50,51^; in this task, participants with repetitive NSSI engaged in persistent, repetitive behavior which no longer met their goals. However, neither sporadic nor repetitive NSSI was associated with impulsive decision making as indexed by the Cambridge Gambling Task or behavioral disinhibition on the Affective Go/No-Go task, even when the emotional valence of distractors was modified.

In addition to the significant compulsivity finding, adolescents with repetitive NSSI demonstrated disadvantageous decision making on the Cambridge Gambling Task—when presented with a set probability of winning (ie, the ratio of colored boxes), they selected the optimal choice less consistently than no-NSSI controls. This suggests that repetitive NSSI is associated with a difficulty in assessing probable outcomes and selecting the optimal long-term decision. This may be present only in those with repetitive NSSI as continued engagement in NSSI despite negative consequences inherently reflects a difficulty in evaluating outcomes. However, disadvantageous decision making was not paired with significantly larger bets, nor faster reaction time, therefore it does not appear to reflect global impulsive decision making.

Our nonsignificant behavioral disinhibition findings are consistent with previous affective Go/No-Go research,^29^ but contrast with past significant results of a similar emotional Stop Signal Task.^28,29^ Both types of laboratory task capture behavioral inhibition, but the critical difference lies in the timing of stimulus presentation. In the Go/No-Go task, participants must withhold a planned response when presented with a distractor stimulus. During the Stop Signal Task, in contrast, participants must inhibit an action which has already begun. This small change is the difference between “action restraint” and “action cancellation.”^52^ While the end result is largely the same—inhibiting a behavioral response—the neural mechanisms underlying the behavior differ.^53^ It may be that only this “action cancellation” pathway is specifically associated with emotion-relevant impulsive engagement in NSSI; impaired action cancellation can create a difficulty in interrupting an emotional reaction, and could explain why people with NSSI struggle to resist acting on NSSI urges once they reach a certain intensity. In support of this assertion, a recent study utilizing both an emotional Stop Signal Task and emotional Go/No-Go Task revealed that NSSI is associated only with impaired negative emotional action cancellation (Stop Signal Task), which likely contributes to difficulty inhibiting negative emotional impulses^29^.

Findings here do fit with the larger body of literature demonstrating that while people with NSSI self-rate their impulsivity as higher, there is little evidence for altered performance on laboratory impulsivity tasks.^25^ People with NSSI may only behave impulsively in the moment when experiencing certain negative emotional states, and therefore perform similarly to healthy controls on behavioral tasks in the absence of such emotions. A comprehensive enquiry into the associations between NSSI and impulsivity and compulsivity phenotypes should include both selfreport and behavioral measures of the constructs^54^.

## Limitations

While our findings suggest valuable new directions in research on neurocognitive functioning and NSSI, this study was a secondary analysis of pre-collected data and therefore hindered by the limitations of the data available. First, participants did not report on NSSI at the time of neurocognitive testing and recency of NSSI was not assessed. We did conduct supplemental sensitivity analyses after removing the n = 18 participants who reported NSSI at age 17, but not at age 14, thus ensuring all individuals in the NSSI group had a history of NSSI at the time of neurocognitive testing. Despite the limited statistical power of these analyses, results replicated those reported here and support the validity of our findings (Table S2).

Second, our classification of repetitive NSSI diverges from the literature standard due to the study being conducted (and questionnaire designed) before publication of the proposed DSM-5 criteria for NSSI disorder, which includes the empirically supported frequency criterion of five or more days per year.^55^ Results would be strengthened if participants had reported a continuous measure of NSSI frequency, as well as additional descriptors of their NSSI engagement.

Third, these neurocognitive tasks cannot capture the real-life context of NSSI, which typically involves strong negative emotions.^2^ Fourth, participants endorsing NSSI were significantly more likely than controls to have a lifetime psychiatric diagnosis, and thus broader symptoms of mental illness may account for the results. While we reduced the effects of this potential confound by controlling for recent depressive symptoms at the time of neurocognitive testing, additional research is needed to elucidate the relationship between NSSI, psychopathology, and cognitive impairments. Fifth, sample size was only moderate, especially for subgroups of sporadic vs repetitive NSSI. Findings need replication in a larger sample. Finally, participants were predominantly of White ethnicity and relatively high socioeconomic status due to the demographic characteristics of Cambridgeshire. As such, further work is needed to confirm the generalizability of the findings to other populations.

## Future directions

Here, we present the first evidence of an association between NSSI and behavioral compulsivity. Additional research is needed to elucidate this relationship. Definitions of “compulsive behaviors” center on the core feeling that one *must* perform the action, or cannot stop the action.^56^ Future studies may wish to consider the lived experience of NSSI as a compulsive behavior and explore how both laboratory and self-report measures relate to the apparent “compulsive” features often reported by people with repetitive NSSI, such as perceived loss of control over frequency and severity, inability to stop self-injuring despite goals to quit, and NSSI urges which are difficult to resist^17,32,33^.

Longitudinal research is needed to evaluate the temporal relationship between NSSI and task performance, in particular to disentangle whether there is a shift from impulsivity to compulsivity as NSSI becomes more chronic, as is seen in substance dependence^51^; whether high compulsivity is a pre-existing risk factor for repetitive NSSI; or whether these neurocognitive traits emerge as a result of repetitive NSSI engagement. Such a distinction is crucial—if there is indeed a shift from impulsivity to compulsivity, this highlights the need for early intervention before the behavior becomes habitual, compulsive, and harder to treat^57^.

Future neurocognitive research must also consider the heterogeneity of NSSI, both within and between individuals. People hurt themselves for a wide range of reasons and typically report multiple different motivators.^3^ While the self-report literature indicates that certain facets of impulsivity are associated with particular NSSI function,^58^ or new onset vs continuation of NSSI,^59^ the same nuanced approach has yet to be undertaken with behavioral neurocognitive measures.

Researchers should investigate how neurocognitive traits interact with other distal (eg, childhood adversity) and proximal (eg, emotion dysregulation) risk factors to contribute to NSSI engagement. Research which considers the nuanced interplay of etiologic factors, motivating and reinforcing pathways, and individual experience can advance our understanding of NSSI and pave the way for evidence-based interventions which alleviate the suffering brought by this harmful behavior. Interventions for other impulsive and compulsive behaviors should be explored for their utility treating repetitive NSSI as well.

## Conclusion

Our findings suggest that lifetime NSSI—particularly repetitive NSSI— is associated with compulsivity and disadvantageous decision making in adolescents. However, we found no evidence of increased impulsivity (emotion-relevant behavioral disinhibition or global impulsive decision making) in the NSSI group. Future research should continue to investigate the relevance of neurocognitive phenotypes in NSSI, in particular the possible association between compulsivity and common features of repetitive NSSI (eg, strong urges, escalation over time, difficulty stopping). NSSI may share neurocognitive etiology with other risky yet rewarding maladaptive behaviors, with potential implications for transdiagnostic interventions.

## Supplementary Material

Table S1

Table S2

## Figures and Tables

**Table 1:** Demographic and clinical characteristics of participants.

	Controls n = 190		Lifetime NSSI n = 50			Sporadic NSSI n = 28		Repetitive NSSI n = 22		
	*M*	*SD*	*M*	*SD*	*p*	*M*	*SD*	*M*	*SD*	*p*
Age (years)	16.38	0.47	16.32	0.40	0.47	16.38	0.41	16.25	0.37	0.23
Full IQ^a^	106.25	16.21	104.70	17.10	0.55	103.50	17.30	106.23	17.12	0.58
MFQ Score^b^	11.39	7.64	18.78	10.29	**<0.001**	16.89	10.09	21.18	10.27	0.15
	*n*	*%*	*n*	*%*	*p*	*n*	*%*	*n*	*%*	*p*
Female	89	46.84	37	74	**0.001**	20	71.43	17	77.27	0.64
White ethnicity	175	92.11	42	84	0.17	23	82.14	19	86.36	0.78
High SES^c^	164	86.32	44	88	0.68	22	78.57	22	100	0.07
Lifetime psychiatric diagnosis	23	12.11	21	42	**<0.001**	8	28.57	13	59.09	**0.045**

Contrasts compared lifetime NSSI vs controls and sporadic NSSI vs repetitive NSSI. Bold indicates significant group differences. Abbreviations: MFQ, Moods and Feelings Questionnaire; SES, socioeconomic status.

a intellectual ability measured via the vocabulary and block design subtests of the Wechsler Intelligence Scale for ChildrenIII, UK version (39).

b Depressive symptoms in the past two weeks measured via the Moods and Feelings Questionnaire (40).

c Socioeconomic status (SES) binarized based on ACORN status as high (‘wealthy’, ‘urban prosperity’, ‘comfortably off) vs low (‘moderate means’, ‘hard pressed’).

**Table 2:** Group Task Performance

	Controls n = 190		Lifetime NSSI n = 50				Sporadic NSSI n = 28				Repetitive NSSI n = 22			
	*M*	*SD*	*M*	*SD*	*p*	*M*	*SD*	*p*	*M*	*SD*	*p*
**Affective Go/No-Go**											
Commission errors											
Total	32.04	18.69	36.16	18.87	0.23	36.64	19.51	0.29	35.55	18.46	0.44
Positive^a^	11.83	6.44	14.00	6.73	0.18^b^	14.18	7.06	0.35^b^	13.77	6.44	0.26^b^
Negative^a^	10.36	6.76	11.94	6.61		11.82	7.09		12.09	6.11	
Neutral^a^	9.56	7.24	10.22	6.93		10.64	6.96		9.68	7.01	
Mean correct latency	495.4	76.8	480.8	80.7	0.44	496.8	76.1	0.74	460.4	83.4	0.08
**Cambridge Gambling Task**											
Proportion of points bet	0.48	0.11	0.46	0.11	0.44	0.47	0.12	0.69	0.46	0.10	0.40
Quality of decision making	0.95	0.07	0.91	0.11	0.015	0.92	0.12	0.14	0.90	0.11	**0.021** ^c^
Response latency	1896.6	606.1	2131.8	852.5	0.18	2055.5	817.3	0.69	2228.9	905.1	0.074
**Probabilistic Reversal Task**											
Perseverations	3.40	2.04	3.88	2.85	0.040	3.50	2.08	0.50	4.38	3.63	**0.006** ^c^
Errors to criterion	2.51	3.89	3.14	4.50	0.14	3.00	4.70	0.53	3.33	4.32	0.11
Probability matching score	0.10	0.16	0.11	0.14	0.58	0.10	0.14	0.92	0.13	0.14	0.27

Multivariate regression analyses included age, sex, intellectual ability, and recent depressive symptoms as covariates. Contrasts compared NSSI groups to controls. Comparisons ofsporadicvs repetitive NSSI did not reveal any significant results.

a Indicates distractor valence.

b NSSI group × valence interaction p-value.

c Significant according to Benjamini-Hochberg procedure.

## Data Availability

Data are available in a public, open access repository. Data are available upon reasonable request. NL had full access to all the data in the study and takes responsibility for the integrity of the data and the accuracy of the data analysis. The data are deposited in the University of Cambridge Data Repository, with the placeholder DOI https://doi.org/10.17863/CAM.65723
