# Supplementary material for: Behavioral measures of impulsivity and compulsivity in adolescents with nonsuicidal self-injury
Source: CNS Spectr. Author manuscript; Available in PMC 2022 Oct 26. (PMC7613746; doi:10.1017/S1092852921000274)
Supplement: Table S1 [file EMS123278-supplement-Table_S1.docx]

Table S1. Analyses relating current mental health (MFQ score) to task performance. All primary analyses included MFQ as a covariate. Bold indicates significance at *p* < 0.05.

| Outcome Variable | Regression type | Coef. | Std. Err. | t- or z-value | *p-*value |
| --- | --- | --- | --- | --- | --- |
| **Affective Go/No-Go**  Total commission errors | Negative binomial | 0.008 | 0.004 | 1.99 | **0.046** |
| Positive commission errors | Negative binomial | 0.01 | 0.004 | 2.49 | **0.013** |
| Negative commission errors | Negative binomial | 0.009 | 0.004 | 2.10 | **0.035** |
| Neutral commission errors | Negative binomial | 0.005 | 0.006 | 0.82 | 0.41 |
| Mean correct latency | Linear | -1121.81 | 530.99 | -2.11 | **0.036** |
| **Cambridge Gambling Task**  Proportion of points bet | Linear | 0.0001 | 0.0008 | 0.14 | 0.89 |
| Quality of decision making | Linear | -0.0007 | 0.0007 | -1.04 | 0.30 |
| Response latency | Linear | -0.00005 | 0.00003 | -2.05 | **0.041** |
| **Probabilistic Reversal Task**  Perseverations | Negative binomial | -0.008 | 0.005 | -1.86 | 0.064 |
| Errors to criterion | Negative binomial | -0.003 | 0.01 | -0.21 | 0.84 |
| Probability matching score | Linear | 0.0002 | 0.001 | 0.17 | 0.86 |
