# Supplementary material for: Behavioral measures of impulsivity and compulsivity in adolescents with nonsuicidal self-injury
Source: CNS Spectr. Author manuscript; Available in PMC 2022 Oct 26. (PMC7613746; doi:10.1017/S1092852921000274)
Supplement: Table S2 [file EMS123278-supplement-Table_S2.docx]

Table S2. Analyses of task performance were repeated after removing the *n* = 18 participants who reported NSSI at age 17 but not at age 14 (i.e. excluding participants who may have first engaged in NSSI after neurocognitive testing). Findings were the same as in primary analyses; compared to no-NSSI controls, participants with lifetime NSSI, and repetitive NSSI specifically, displayed significantly lower quality of decision making on the Cambridge Gambling Task and more perseverative errors on the Probabilistic Reversal Task.

| Affective Go/No-Go | Controls  *n* = 185 | | Lifetime NSSI  *n* = 32 | | | Sporadic NSSI  *n* = 17 | | | Repetitive NSSI  *n* = 15 | | |
| --- | --- | --- | --- | --- | --- | --- | --- | --- | --- | --- | --- |
|  | *M* | *SD* | *M* | *SD* | *p* | *M* | *SD* | *p* | *M* | *SD* | *p* |
| Commission Errors |  |  |  |  |  |  |  |  |  |  |  |
| Total | 32.04 | 18.69 | 37.84 | 20.86 | 0.17 | 36.06 | 22.01 | 0.55 | 39.87 | 20.04 | 0.12 |
| Positive ^a^ | 11.83 | 6.44 | 14.41 | 7.21 | 0.18 | 13.29 | 7.61 | 0.72 | 15.67 | 6.77 | 0.08 |
| Negative ^a^ | 10.36 | 6.76 | 12.50 | 6.80 |  | 11.53 | 7.10 |  | 13.60 | 6.51 |  |
| Neutral ^a^ | 9.56 | 7.24 | 10.94 | 7.77 |  | 11.24 | 8.13 |  | 10.6 | 7.60 |  |
| Mean Correct Latency | 495.39 | 76.81 | 471.48 | 83.67 | 0.26 | 487.86 | 81.79 | 0.94 | 452.91 | 84.62 | 0.06 |
| Cambridge Gambling Task | Controls  *n* = 190 | | Lifetime NSSI  *n* = 32 | | | Sporadic NSSI  *n* = 17 | | | Repetitive NSSI  *n* = 15 | | |
|  | *M* | *SD* | *M* | *SD* | *p* | *M* | *SD* | *p* | *M* | *SD* | *p* |
| Proportion of    Points Bet | 0.48 | 0.11 | 0.45 | 0.10 | 0.26 | 0.46 | 0.10 | 0.60 | 0.44 | 0.10 | 0.22 |
| Quality of  Decision Making | 0.95 | 0.07 | 0.90 | 0.12 | **0.015** | 0.92 | 0.12 | 0.217 | 0.88 | 0.13 | **0.013** |
| Response  Latency | 1896.62 | 606.14 | 2144.57 | 872.27 | 0.31 | 2062.51 | 842.22 | 0.96 | 2237.57 | 925.55 | 0.12 |
| Probabilistic Reversal Task | Controls  *n* = 185 | | Lifetime NSSI  *n* = 31 | | | Sporadic NSSI  *n* = 17 | | | Repetitive NSSI  *n* = 14 | | |
|  | *M* | *SD* | *M* | *SD* | *p* | *M* | *SD* | *p* | *M* | *SD* | *p* |
| Perseverations | 3.40 | 2.04 | 4.06 | 3.39 | **0.016** | 3.65 | 2.34 | 0.25 | 4.57 | 4.38 | **0.009** |
| Errors to  Criterion | 2.51 | 3.89 | 2.74 | 3.80 | 0.21 | 1.59 | 1.91 | 0.65 | 4.14 | 5.01 | 0.053 |
| Probability    Matching Score | 0.10 | 0.16 | 0.10 | 0.13 | 0.81 | 0.06 | 0.07 | 0.10 | 0.16 | 0.16 | 0.17 |
